# Human Platelets in Intravenous Fluids Probed by Raman Tweezers Spectroscopy

**DOI:** 10.1021/acs.analchem.4c05095

**Published:** 2025-03-27

**Authors:** Mithun Nelliat, Ganesh Mohan, Shamee Shastry, Jijo Lukose, Murukeshan Vadakke Matham, Santhosh Chidangil

**Affiliations:** † Centre of Excellence for Biophotonics, Manipal Institute of Applied Physics, Manipal Academy of Higher Education, Manipal, Karnataka-576104, India; ‡ Department of Immunohematology and Blood Transfusion, Kasturba Medical College Manipal, Manipal Academy of Higher Education, Manipal, Karnataka-576104, India; § Manipal Institute of Applied Physics, Manipal Academy of Higher Education, Manipal, Karnataka-576104, India; ∥ Centre for Optical and Laser Engineering, School of Mechanical Aerospace Engineering, Nanyang Technological University, 50 Nanyang Avenue, 639798, Singapore

## Abstract

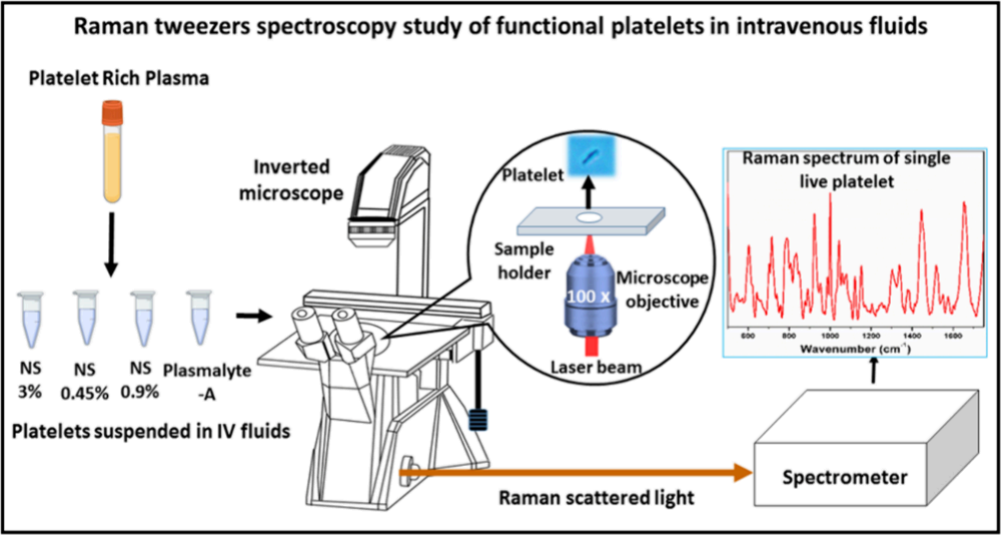

Intravenous fluids (IVs) play crucial roles in postblood transfusions. It is frequently used for cell washing in some cases, before transfusions, to prevent allergic reactions in patients with hypersensitivity to plasma proteins and also to reduce risk in certain situations, such as acute lung injury due to transfusion-related shock, patients with impaired immune systems, and pediatric patients. Other than cell washing, intravenous fluid therapy is an essential medical practice in hospitals. Despite their widespread use, the effects of IV fluids on platelet function have not been thoroughly explored. The present study investigates the interaction of live platelets with various IV fluids, such as normal saline, hypertonic saline, hypotonic saline, and plasmalyte-A for the first time using a laboratory assembled Raman Tweezers system. Platelet additive solution is considered as the control for the experiment. The result shows that the platelets treated with intravenous (IV) fluids had higher peak intensities in the phospholipid Raman marker bands, corresponding to heightened surface expression of phospholipids and proteins, which are indicative of platelet activation. The morphological changes such as filopodia formations on platelets and shape change from discoid to spherical form support the above said findings. While platelet activation has great significance for hemostasis, needless activation can cause harmful effects on people suffering from various health conditions, such as cardiovascular diseases.

## Introduction

Platelets, also called thrombocytes, are an important blood component for clot formation. In healthy humans, platelets have a lifespan of approximately 7 to 10 days.^1^ Platelets remain inactive and exhibit a discoid shape within the body. However, upon activation, their morphology transforms, leading to the development of small protrusions on their surface, known as filopodia.^2^ During this activation, significant alterations occur in the platelets’ structure and biochemical characteristics.^3^ The single live platelet study under different conditions will help to understand more about the active and inactive platelets and their interactions.^4^ The increased platelet activation will lead to the formation of thrombus and ischemic injury to the organs, especially the heart (myocardial infarction), brain (stroke), limbs, and intestine.^5−7^ In the realm of medicine, intravenous fluid therapy is a crucial procedure for saving lives. Trauma patients and individuals with other health conditions, such as vomiting or diarrhea, can lead to bodily fluid depletion.^8−10^ The blood cells were in direct contact with the intravenous fluids throughout the fluid administration. The volume of administered IVs and the composition of the fluid depends on the patient’s need.^11^ Several research groups have conducted clinical studies to examine how intravenous fluids affect platelets and other blood components. Cell washing is a standard clinical practice carried out before transfusions to ensure the safety of the recipient.^12^ Typically, this procedure involves using a normal saline solution with a concentration of 0.9%. However, the potential negative impact of normal saline on washed cells has been investigated by different research groups.^13,14^ Majed et al. experimented on platelets and RBCs to examine how plasmalyte-A and ordinary saline (0.9%) affect these cells.^15^ Both normal saline and plasmalyte-A were used to wash platelets and RBCs, and they found that washed RBCs with normal saline increased the hemolysis rate at the same time the plasmalyte-A did not have an adverse effect on the washed RBCs, while washing platelets with plasmalyte-A did not decrease platelet function. Jill et al. also found that the cells washing with plasmalyte-A showed good platelet function and less RBC hemolysis during storage.^16^ The effect of platelets in hypertonic saline, hypotonic saline, and plasmalyte-A was studied and reported in this paper using the Raman Tweezers Spectroscopy technique.

Raman spectroscopy has been recognized as a versatile analytical method for examining samples at the molecular level.^17^ The minimum or no sample preparation, nondestructive, good sensitivity and specificity are the significant advantages of the Raman spectroscopy technique.^18^ When monochromatic light interacts with matter, it experiences various processes, such as absorption, emission, refraction, reflection, and scattering. Raman spectroscopy is rooted in inelastic light scattering.^19^ Inelastic scattering, also known as Raman scattering, occurs when the scattered light has an altered frequency compared with the incident light. Elastic scattering, also known as Rayleigh scattering, occurs when the scattered light keeps the frequency the same as that of incident light.^20^ Sir C.V. Raman showed that the energy of photons undergoing inelastic scattering can serve as a unique “signature” of the material from which the scattering occurs.^21^ So, the Raman spectroscopy has been accepted as a versatile method in biomedical research, chemical analysis, pharmaceutical sciences, and many other fields.^22,23^

The Raman spectroscopy technique, with the combination of optical tweezers, is an exceptional tool for studying single live cells in their physiological condition.^24,25^ This technique was extensively used for the investigation of platelets, red blood cells (RBCs), white blood cells (WBCs), bacteria, cancer cells, etc.^26−28^ The primary benefit of integrating optical tweezers with Raman spectroscopy lies in its ability to examine live cells, specifically platelets, by individually capturing them in their physiological environment.^29^ Live platelets exhibit Brownian motion within the suspension medium, making it challenging to obtain Raman spectra from these moving cells. Therefore, the incorporation of an optical tweezers system is essential for immobilizing the cells. Additionally, this technique is advantageous, as it is label-free, eliminating the need for external agents or chemical fixatives to stabilize the cells. Given that platelets are highly sensitive blood components, any adverse impact could trigger their transition from their inactive state to the active state. Chemical fixation would stop all metabolic activities, rendering the platelets nonviable for live investigations, and the chemicals used could further adversely affect them.^30^ Consequently, the combination of optical tweezers and Raman spectroscopy presents a superior method for studying single live platelets.

The consequences of normal saline on live RBCs using Raman tweezers spectroscopy was studied by Jijo et al. The deoxygenation of RBCs in normal saline was observed from this experiment.^31^ When red blood cells undergo deoxygenation, the oxygen molecules bound to the iron atom within the porphyrin compound of heme will detach, altering the chemical structure. This structural modification is reflected in the intensity changes occurring to the bands at 565, 1222, 1561, and 1636 cm^–1^. The interaction of RBCs with crystalloid and colloidal intravenous fluids also shows oxy-deoxy changes in their Raman bands.^32,33^ Compared to blood plasma, RBCs suspended in crystalloid fluids had a lower oxyhemoglobin status, as indicated by the ratio of the oxygenation/deoxygenation markers’ Raman band intensity at the spin marker region and methine deformation region. Only limited studies were reported on a single live platelet using the optical tweezers Raman spectroscopy technique. One of the few studies conducted on platelets was an investigation of live platelets from the rat model as part of their research on Alzheimer’s disease.^34^

The current study discusses the interaction of platelets with different intravenous fluids probed by the Raman tweezers spectroscopy technique. The platelets suspended in PAS are considered the control sample for the current work. The study also addresses the changes in Raman bands associated with platelet activation in intravenous fluids. Furthermore, it included the examination of morphological alterations in platelets when they were exposed to various intravenous fluids using microscopic images. The results presented here were the outcome of the experiments conducted under in vitro condition. In order to perform in vivo measurements, one must address significant challenges. During in vivo investigations, there would be interference/involvement of additional blood components, including red and white blood cells, which are comparatively large in size, while platelets size range from 2 to 3 μm. Therefore, capturing and recording Raman spectra from individual platelets would be exceedingly complex. Furthermore, the continuous flow of blood through the blood vessels would necessitate a substantial trapping force to immobilize the target cell, potentially causing damage to both the skin and the blood components; thereby, performing an in vivo study is impractical with the existing setup.

## Materials and Methods

**Figure 1:**
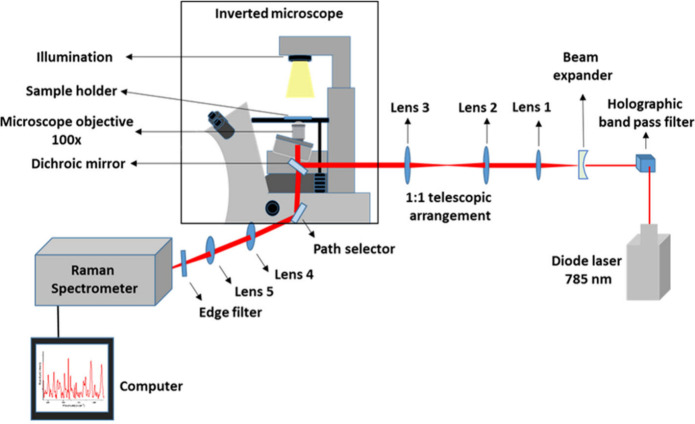
Schematic representation of the lab-assembled optical tweezers Raman spectroscopy setup.

**Figure 2:**
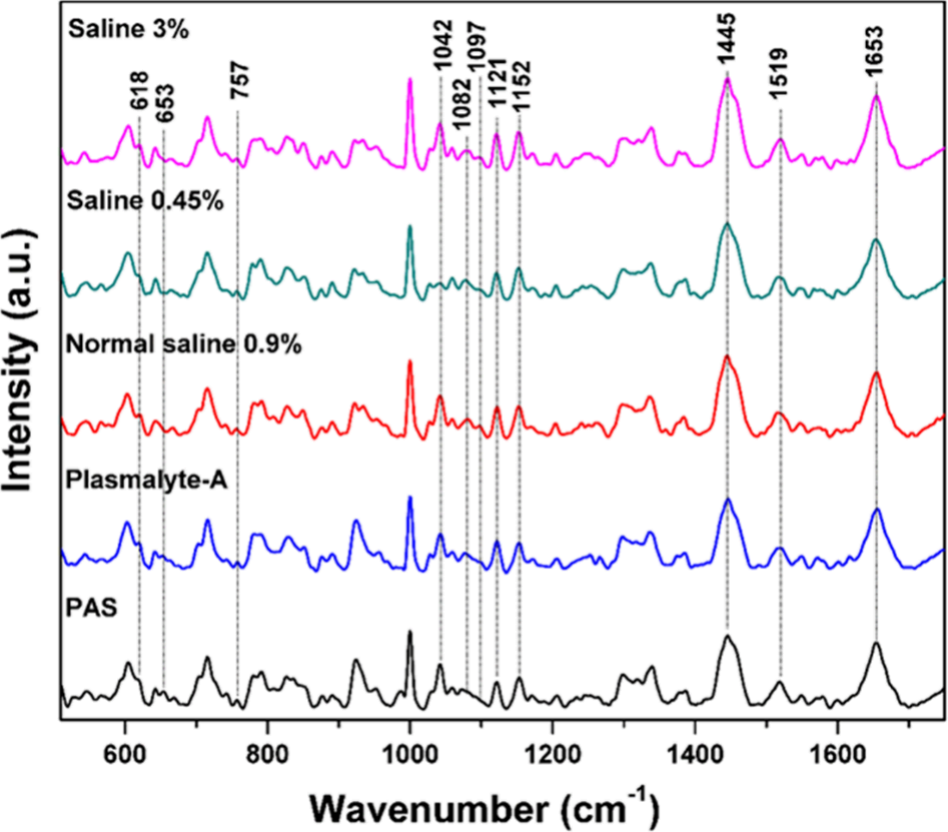
Raman spectra of platelets suspended in a platelet additive solution (PAS) and intravenous fluids.

The experiments were conducted with a lab-assembled Raman Tweezer setup, employing a 785 nm diode laser (Star Bright laser) for the trapping and probing of the cells. The schematic of Raman tweezers setup was depicted in Figure 1. A holographic bandpass filter was placed to eliminate wavelengths apart from 785 nm. The trapping of a live cell necessitates a highly focused laser beam; hence, a 100× microscope objective was employed. An inverted microscope was used for the setup, offering exceptional stability for trapping. The laser beam was able to fully fill the back aperture of the microscope objective by using an expander to increase the beam diameter. This, in turn, resulted in a more tightly focused laser beam on the sample plane, thereby enhancing the trapping force. The telescopic arrangement with a 1:1 ratio enables precise control of the focused beam on the sample plane. An edge filter was used to eliminate the elastically scattered light. The scattered light from the sample passes through the edge filter before being focused into the spectrometer (spectrometer has a 1200 grooves/mm grating blazed at 750 nm) with liquid nitrogen-cooled CCD. To mitigate noise in the Raman spectra obtained from a single platelet, the accumulation number for each spectrum was set to 2. The spectra illustrated in Figure 2 represent the average of 25 individual spectra. From each intravenous fluid, 25 individual platelets were optically trapped, and Raman spectra were recorded with the accumulation number as 2 for each spectrum.

The present setup involved a single laser for optically trapping and probing the platelets, which were suspended in different intravenous fluids possessing distinct tonicity. The platelet rich plasma were collected from blood bank, KMC Manipal with the ethical approval obtained from Kasturba Medical College and Kasturba Hospital Institutional Ethics Committee (IEC: 68/2018). The spectra were recorded with a laser power of 10 mW (on the sample), a 60 s acquisition time, and the number of accumulation as 2. In this study, 5 μL of platelet-rich plasma was suspended in 2 mL of various intravenous fluids. Subsequently, 300 μL of the prepared sample was transferred into a fused-silica-based sample holder for Micro-Raman measurements. The detailed description about the separation of platelets from whole blood is given in the Supporting Information. The intravenous fluids utilized in the study comprise normal saline 0.9% (NS 0.9%), hypotonic saline 0.45% (NS 0.45%), hypertonic saline 3% (NS 3%), plasmalyte-A, and a platelet additive solution (PAS) as the control.

## Results and Discussion

### Raman Spectra of Platelets Treated with IV Fluids

The Raman spectra of a single, live platelet suspended in platelet additive solution (PAS) and intravenous fluids are given in Figure 2. The platelets in the PAS serve as the control group for this investigation. Notably, the Raman spectra of platelets in intravenous fluids exhibit certain variations in Raman band intensity compared to those in PAS. The significant alterations in Raman bands are indicated in Figure 2. Most labeled bands arise from the phospholipids, which are mainly found within the platelet membrane. The platelet membrane structure is composed of a characteristic phospholipid bilayer.^35,36^ The platelet possesses a distinctive characteristic in which its plasma membrane encompasses an intricate network of multiple invaginations into the interior of the platelet. These indentations are linked to the outside environment via tiny openings, forming the open canalicular system.^37^ This system serves as the route for the movement of substances into the cell and the release of granular products generated during platelet activation.^38,39^ Aminophospholipids such as phosphatidylserine and phosphatidylethanolamine are abundant in the plasma membrane, specifically located in the inner leaflet.^40,41^ When platelets are activated, these aminophospholipids move to the outer leaflet of the membrane.^42^ As a result, the concentration of these aminophospholipids in the outer leaflet increases during platelet activation.^43,44^ The Raman spectra depicted in Figure 4 exhibit an increased intensity of phospholipid bands (757, 1082, 1097, 1121, 1436, 1445, and 1455 cm^–1^) of platelets suspended in intravenous fluids, particularly in saline solutions. This may be attributed to the accelerated activation rate of platelets in saline solutions. Furthermore, the microscopic images demonstrate the increased level of activation of platelets in hypertonic and hypotonic saline solutions. The Raman spectra of blood plasma and platelets suspended in plasma are given in the Supporting Information, Figure 1S, which shows the overlapping of certain intense plasma peaks with platelet peaks. Similarly, the Raman spectra of all the intravenous fluids used for the current work are also provided in Figure 2S, which shows that the IV fluids do not have any characteristic Raman peaks overlapping with platelet spectra. The Raman band assignments are given in the Supporting Information, Table 1S.

### Lipid Bands

**Figure 3:**
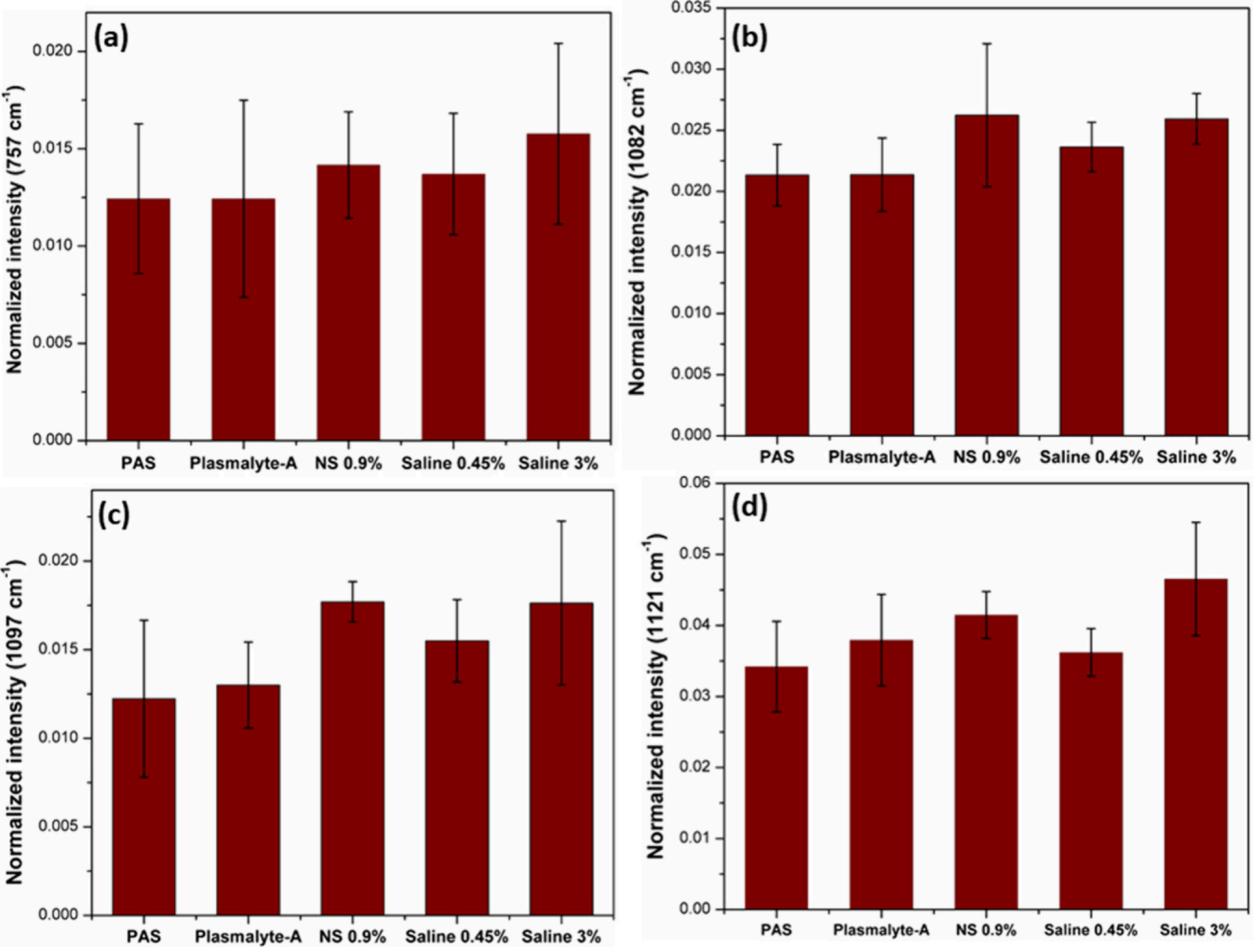
Bar plot showing intensity variations for the phospholipid bands: (a) 757, (b) 1082, (c) 1097, and (d) 1121 cm^–1^.

**Figure 4:**
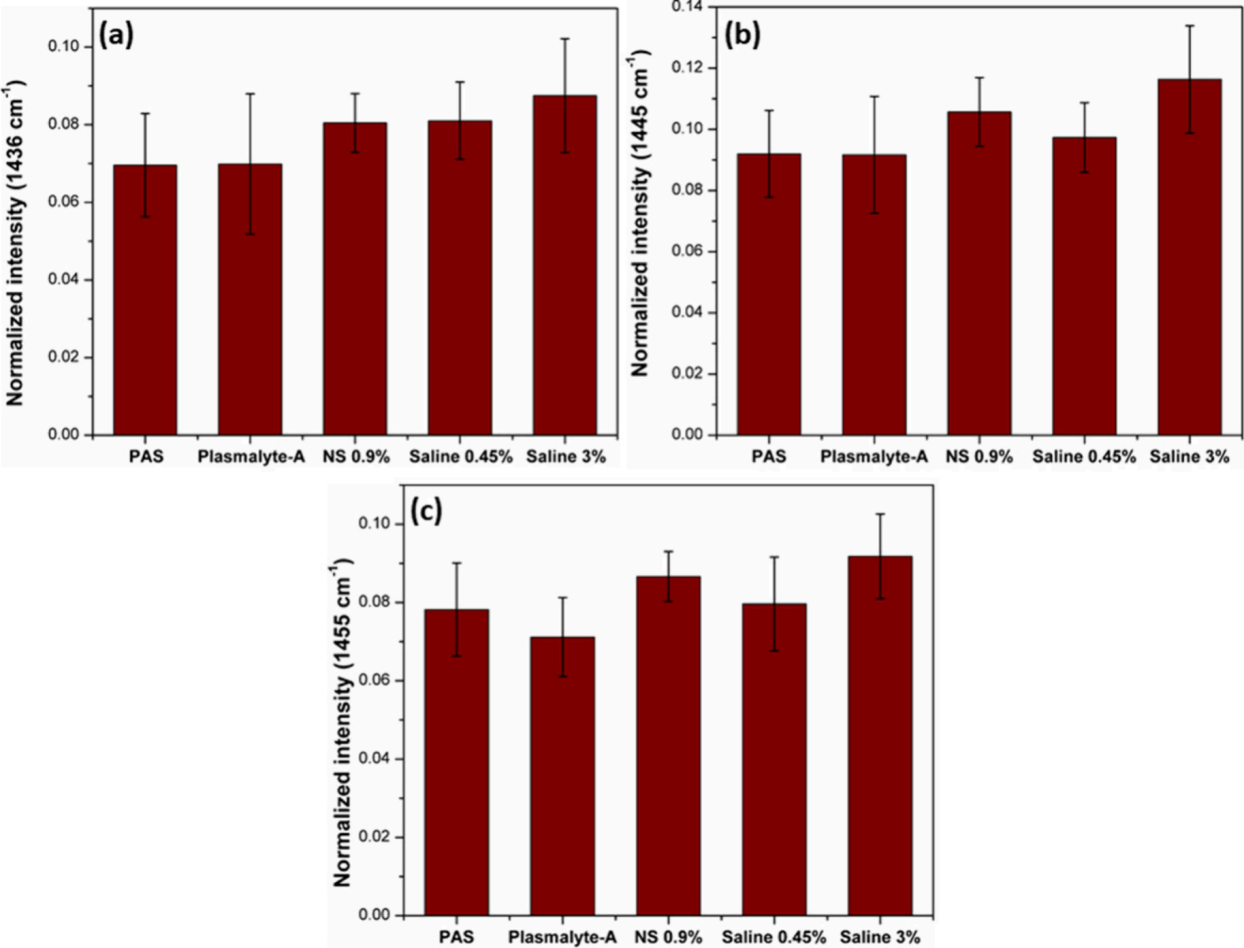
Bar plot showing intensity variations for the phospholipid bands: (a) 1436, (b) 1445, and 1455 cm^–1^.

**Figure 5:**
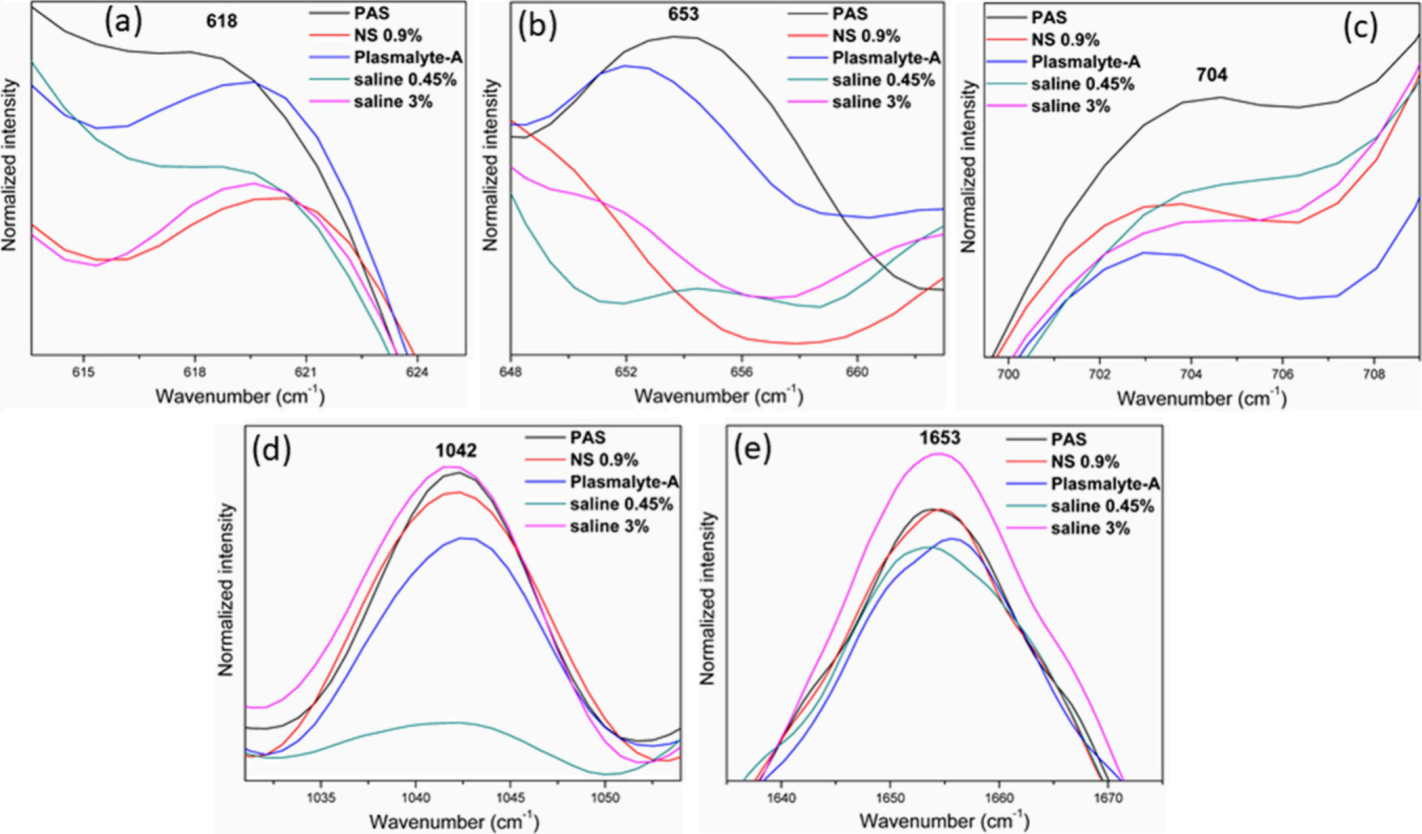
Expanded bands in the Raman spectra of platelets suspended in different IV fluids.

**Figure 6:**
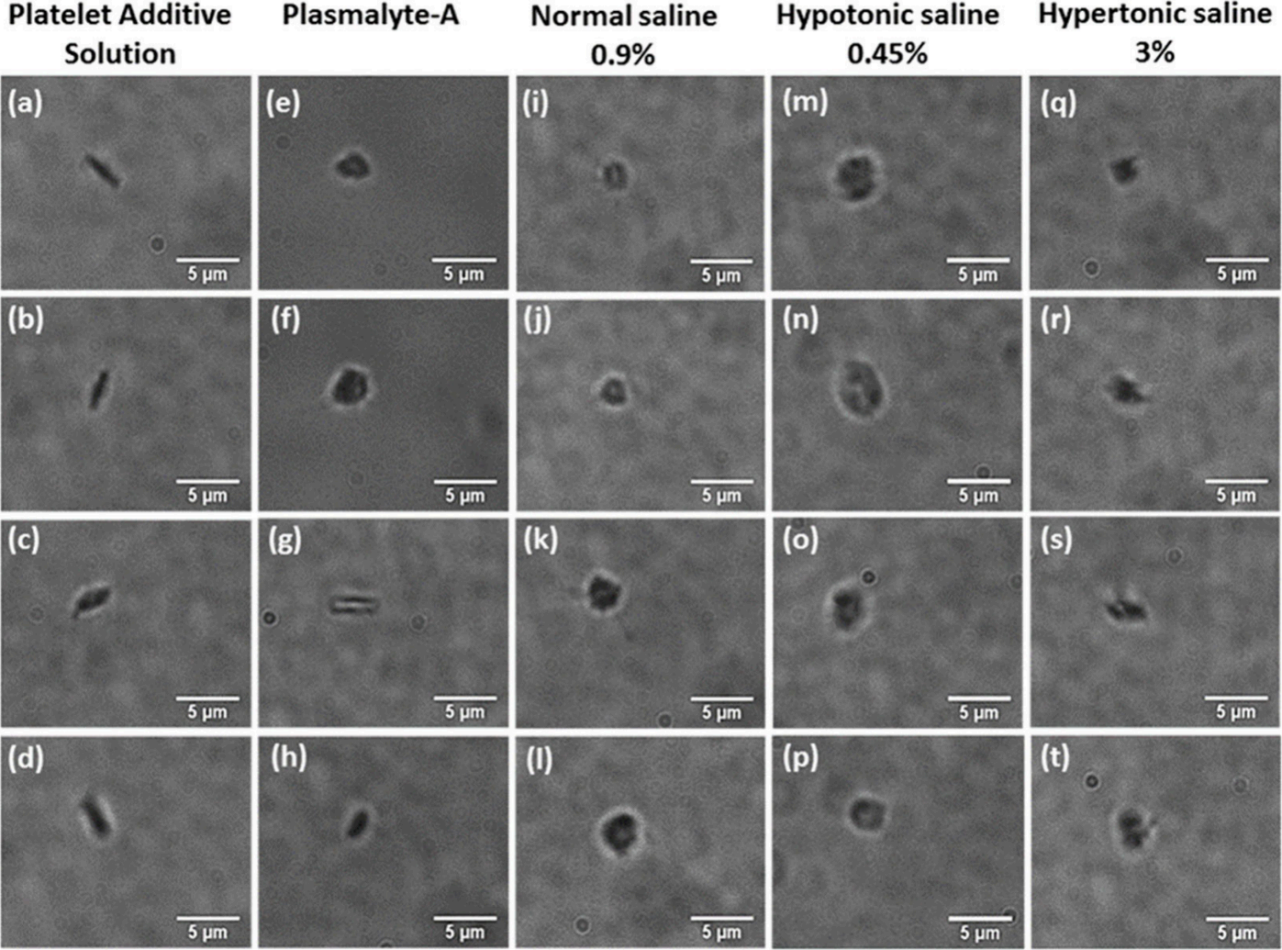
Microscopic images of platelets suspended in platelet additive solution and different intravenous fluids.

The formation of filopodia in these fluids is apparent from the microscopic images in Figure 6. The Raman bands of platelets suspended in hypertonic, hypotonic, and normal saline exhibit higher intensity at 757 cm^–1^ (C–N stretch of phospholipid), 1082 cm^–1^ (C–N stretch of phospholipid), 1097 cm^–1^ (C–C gauche stretch of phospholipid), 1121 cm^–1^ (C–C trans stretch of phospholipid), 1436 cm^–1^ (CH_2_ bending of phospholipid), 1445 cm^–1^ (CH_2_ bending), and 1455 cm^–1^ (CH_2_ bending of lipid) in comparison to PAS and plasmalyte-A. Figures 3 and 4 display the bar plots depicting the intensities of the Raman bands of the phospholipid at 757, 1082, 1097, 1121, 1436, 1445, and 1455 cm^–1^. The increase in intensity of these bands serves as clear evidence of an increased activation rate of platelets in saline solutions. The area under the curve of the figure for phospholipid bands and the corresponding bar plots are given in the Supporting Information (Figures 3S–6S).

### Proteins and Amino Acid Bands

**Figure 7:**
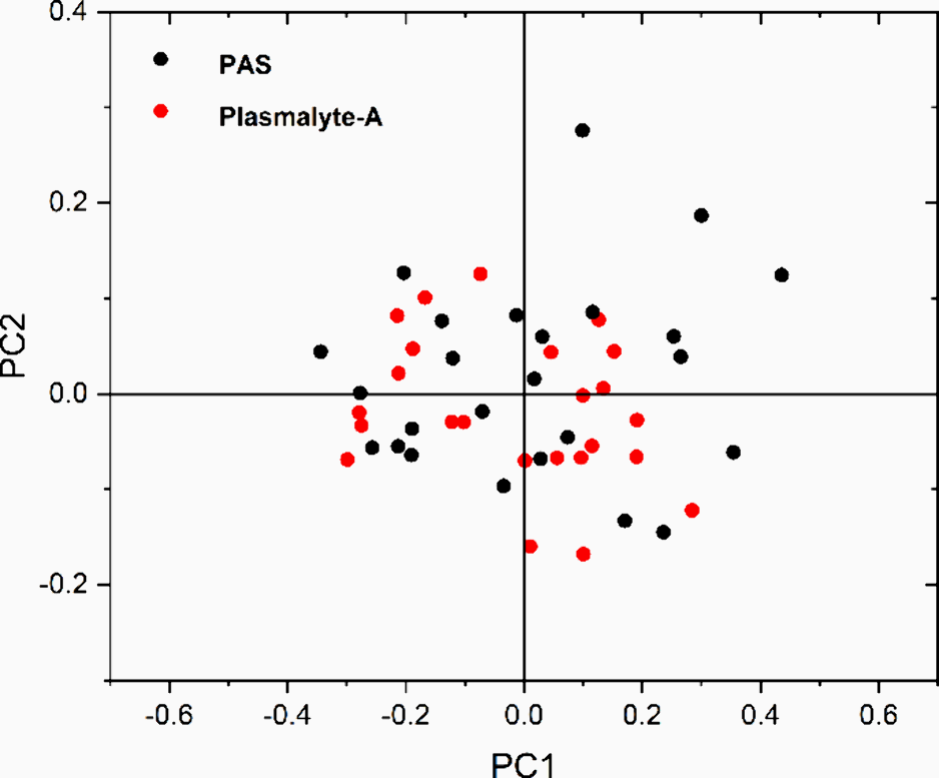
PCA score plot of Raman spectra of platelets in PAS and plasmalyte-A.

Specific regions of Raman spectra featuring platelets suspended in different intravenous fluids and PAS are displayed in Figure 5. Notably, the intensity of the 618 cm^–1^ (C–C twist) band of phenylalanine is higher in PAS and Plasmalyte-A. Conversely, platelets in hypotonic saline, hypertonic saline, and normal saline exhibit a reduced intensity at the 618 cm^–1^ band. The Raman band at 653 cm^–1^ from the tyrosine also exhibits increased intensity in PAS and Plasmalyte-A, while it is comparatively less intense in all three saline solutions. Similarly, the band at 704 cm^–1^, associated with the C–S stretching vibration, has a higher intensity in PAS, but a lower intensity in all four intravenous fluids. In addition, the 1042 cm^–1^ band, which arises from the C–N stretch, and the 1653 cm^–1^ band, due to the C=C stretch of cholesterol, and amide I, exhibit an intensity increase in hypertonic saline but decreased intensity in Plasmalyte-A and less in hypotonic saline. The deconvolution of the amide I band (1600–1700 cm^–1^) and its detailed description are provided in the Supporting Information (Figure 7S). Notably, when platelets are suspended in hypotonic saline, there is a noticeable decrease in the intensity of the 1042 cm^–1^ band. The alterations observed in these Raman spectral features result due to the adverse effects of IV fluids, and this leads to the activation of platelets. The platelet activation manifests a range of morphological and chemical changes.

### Microscopic Images of Platelets Suspended in IV Fluids

The platelets suspended in different intravenous fluids exhibited distinct morphological changes. Figure 6 displays microscopic images of platelets in various solutions, including PAS, plasmalyte-A, NS 0.9%, 3% NS, and NS 0.45%. Platelets suspended in PAS exhibit an average diameter ranging from 2.5 to 3 μm. During activation, the morphology of the platelets transforms from a discoid to a spherical shape. This transformation is also accompanied by the formation of small projections known as filopodia on the platelet surface. The presence of filopodia is the primary indicator of platelet activation. In PAS, no significant difference was observed in platelet size and shape, and the majority of the platelets maintain their normal discoid shape. The diameter of platelets in plasmalyte-A and NS 0.9% is almost similar to that of platelets in PAS. In hypertonic saline, the platelets undergo a reduction in diameter and a transformation from a discoid shape to a shrunken form. The shrunken platelets have a size that approximately ranges from 1.5 to 2 μm. Conversely, in hypotonic saline, the discoid platelets adopt a spherical form and experience an increase in size. The average size ranges from approximately 3 to 4 μm.

In hypertonic saline, platelet shrinkage and disruption of the typical discoid shape were observed. Similarly, treating platelets with normal saline led to enhanced activation, characterized by a higher occurrence of filopodia formation. In their investigation of preserved platelets, Baldini et al. discovered a noticeable reduction in platelet viability when the platelets were stored in a saline solution; this study was also an example of the adverse effect of saline on platelets.^45^Figure 6a–d illustrates microscopic images of platelets suspended in PAS. Figure 6a,b,d shows inactive platelets with a discoid shape. In the case of plasmalyte-A, the platelet remains inactive (Figure 6g), but active platelets were also present in plasmalyte A (Figure 6e,f,h). However, for all other suspension media, nearly all of the platelets appear to be in an activated state. In hypotonic saline, the platelets bulged, resulting in a spherical shape and increased filopodia formation on their surface. The activation of platelets in this context may be attributed to variations in the pH and osmolarity of the suspension media. Researchers find that extreme pH values, like pH values less than 6.4 and greater than 7.6, will cause the transformation of inactive platelets to their active form.^46,47^ The size of the platelets in hypotonic saline is significantly larger, surpassing 3 μm, which exceeds the usual platelet size. Conversely, the platelet size in hypertonic saline is notably smaller than that in a normal platelet size. Within the blood bank, it is imperative to exercise control over multiple factors when storing platelets. Temperature and pH are particularly influential factors, as a decrease in pH can have detrimental effects on the viability of platelets.^48,49^ The pH values of saline solutions vary depending on the type. For instance, normal saline 0.9% has a pH value of approximately 5.23, while hypertonic saline and hypotonic saline have pH values of 6.22 and 5.52, respectively. The pH values of PAS and plasmalyte-A are approximately 7.5 and 7.4, respectively, which closely resemble the pH values of normal blood. This similarity may explain the increased activation of platelets when suspended in saline solutions compared to PAS and plasmalyte-A. However, in the case of PAS and plasmalyte-A, some platelets are active and some remain inactive, possibly due to the ambient temperature of 20 °C. In platelet-rich plasma, the platelets have varying ages, with some being newly formed and others possibly being 2, 3, or more days old. This means that the viability of these platelets can differ under ambient temperature and other environmental factors. This variability might explain why certain platelets appear to be active in solutions such as PAS and Plasmalyte-A.

### Principal Component Analysis (PCA)

Figure 7 displays the PCA score plot for platelets treated with PAS and plasmalyte-A. The score plot does not exhibit a distinct separation between the two samples. Instead, PAS and plasmalyte-A are clustered, suggesting that the disparities in the Raman spectra of platelets suspended in these two solutions are minimal.

**Figure 8:**
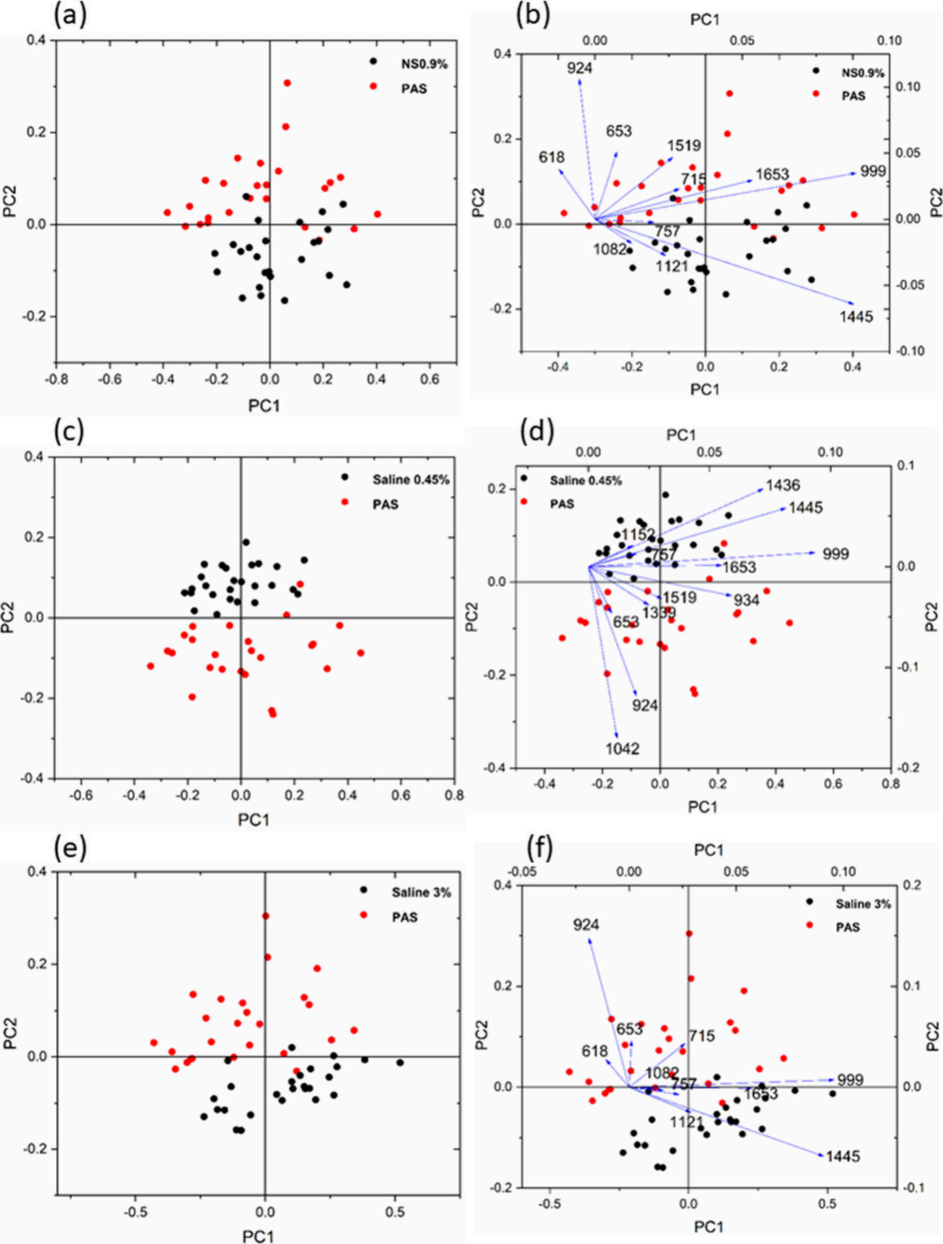
PCA score plots (a, c, and e) and biplots (b, d, and f) of Raman spectra of platelets in different IV fluids.

Figure 8 presents the PCA score plots and biplots to analyze platelets suspended in IV fluids. The score plot demonstrates distinct clustering of platelets treated with PAS and saline solutions. Both the score plot and biplot employ the principle components PC1 and PC2. The loadings (vectors) depicted in the biplots correspond to the variables, specifically, the frequencies measured in wavenumber units, significantly influencing the differentiation of the samples. As a result, the vectors in the biplots correspond to the peaks observed in the Raman spectra, which play a crucial role in differentiating and classifying the samples. The score plot reveals distinctive clusters for PAS and normal saline 0.9% (Figure 8a,b). The biplot highlights the primary Raman bands that contribute to this clustering, namely, 618, 653, 715, 924, 999, and 1653 cm^–1^. These vectors consistently align with the PAS-clustered region. In contrast, the bands at 1082, 1121, and 1445 cm^–1^ are oriented toward the clustered region representing normal saline. It is essential to consider that the bands mentioned are derived from the phospholipids in the platelet membrane upon activation. Similarly, in the case of hypotonic saline and PAS (Figure 8c,d), the clustering is primarily caused by the Raman bands at 1042, 924, 653, 1339, 1519, and 934 cm^–1^, all of which indicate the PAS-clustered region.

It has been evident that the orientation of the Raman bands at 1436, 1445, 999, 1152, and 1653 cm^–1^ is toward the hypotonic saline-treated sample cluster. The clustering observed in PAS and hypertonic saline (Figure 8e,f) is due to the Raman bands at 618, 653, 715, and 924 cm^–1^, all of which indicate the location of the PAS-clustered area. The bands at 757, 1121, 1653, and 1445 cm^–1^ are directed toward the region where hypertonic saline samples cluster. The biplot analysis indicates that the intensity changes of phospholipid bands are the primary factor responsible for the clustering of platelets suspended in PAS compared to other saline solutions.

## Conclusion

The present work investigated the impact of intravenous fluids with different tonicities on human platelets using the Raman tweezers spectroscopy technique. Platelet activation typically occurs due to damage to blood vessels and bleeding. During platelet activation, some phospholipids, particularly phosphatidylethanolamine and phosphatidylserine, usually found on the inner side of the platelet plasma membrane, relocate to the outer side of the membrane. The Raman study shows a decrease in platelet activation within the PAS. Platelet activation was more pronounced in hypertonic, normal, and hypotonic saline solutions. The intensity of specific Raman marker bands associated with phospholipids was higher in IV fluids, indicating a higher rate of platelet activation. Additionally, microscopic images of platelets suspended in these fluids revealed filopodia formation on the surface of activated platelets. The platelets transform their shapes from discoid to spherical forms. Plasmalyte-A exhibits a lower level of platelet activation compared with the saline solutions. The intensity of Raman bands attributed to phospholipids present in platelets suspended in Plasmalyte-A displays minimal variation compared to that in PAS. Traditionally, platelet washing has been conducted using saline solution with a concentration of 0.9%. Nevertheless, the current research has revealed the detrimental impacts of normal saline-based IV fluids on platelets. PAS or plasmalyte-A can be used as an alternative to enhance platelet quality. However, further clinical studies are required to strengthen the outcome of the present observation, which may lead to valuable insights for safer cell washing and intravenous fluid administration.

## Supporting Information Available

Raman spectra of platelets suspended in blood plasma; Raman spectra of intravenous fluids; Raman band assignments for active and inactive platelets and their differences; Platelet separation procedure from whole blood; Platelet activation mechanism; Area under the curve for phospholipid bands; Deconvolution of the amide I band (PDF)
