# Supplementary material for: Human Platelets in Intravenous Fluids Probed by Raman Tweezers Spectroscopy
Source: Anal Chem. 2025 Mar 27;97(13):7028–36. doi: 10.1021/acs.analchem.4c05095 (PMC11983373; doi:10.1021/acs.analchem.4c05095)
Supplement: Supplementary file 1 — ac4c05095_si_001.pdf [file ac4c05095_si_001.pdf]

Supporting Information

Human Platelets in Intravenous Fluids Probed by Raman Tweezers Spectroscopy

Mithun Nelliat[1], Ganesh Mohan[2], Shamee Shastry[2], Jijo Lukose[3], Murukeshan Vadakke Matham[4], Santhosh Chidangil[1]*

1. Centre of Excellence for Biophotonics, Manipal Institute of Applied Physics, Manipal Academy of Higher Education, Manipal, Karnataka-576104, India
2. Department of Immunohematology and Blood Transfusion, Kasturba Medical College Manipal, Manipal Academy of Higher Education, Manipal, Karnataka-576104, India
3. Manipal Institute of Applied Physics, Manipal Academy of Higher Education, Manipal, Karnataka-576104, India
4. Centre for Optical and Laser Engineering, School of Mechanical Aerospace Engineering, Nanyang Technological University, 50 Nanyang Avenue, 639798, Singapore

Corresponding author email:* santhosh.cls@manipal.edu

Table of Contents

Sl. No.	Topic	Page
**1**	Platelets suspended in blood plasma	S-2
**2**	Raman spectra of Intravenous fluids	S-3
**3**	Raman band assignments	S-4
**4**	Platelet separation procedure	S-5
**5**	Platelet activation mechanism	S-5
**6**	Area under the curve	S-6
**7**	The deconvolution of Amide I band (1600-1700 cm^{-1})	S-8
**8**	References	S-10

1. Platelets suspended in blood plasma

The focus of the present investigation is to probe the consequences of intravenous fluids on individual live platelets. The Raman spectra were acquired by trapping the single live platelet using a tightly focused laser beam. The reference sample (control) for the investigation was platelets suspended in platelet additive solution (PAS), which is recognized as one of the platelet storage media used in the Blood Transfusion Centers [1]. Several research groups have done comparative analysis of the blood plasma with PAS as the platelet storage medium. The consistent findings support PAS as the preferred option, as it prolongs the storage duration of platelets and preserves their quality [2, 3]. Figure 1S shows the Raman spectra of blood plasma alone and the single live platelets in plasma. The prominent high-intensity bands (999 cm^{-1}, 1299 cm^{-1}, 1445 cm^{-1}, and 1653 cm^{-1}) observed in the spectra of both samples, suggesting that the platelet signals are always influenced by the presence of blood plasma. So, obtaining distinct Raman signatures for live platelets suspended in plasma was hindered due to the dominant presence of Raman bands originating from the blood plasma. Based on the previous observations [1, 2], and the spectral interference from blood plasma, the platelet additive solution (PAS) was selected as the control medium for this study.

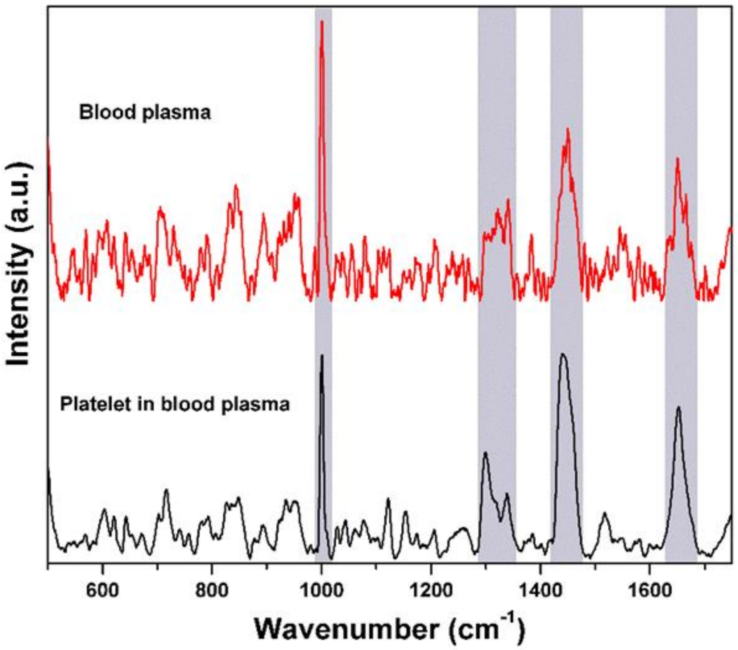

Figure 1S. The Micro-Raman spectrum of blood plasma and platelets suspended in blood plasma.

2. Raman spectra of Intravenous fluids

Raman spectra of intravenous fluids were recorded (Figure 2S) to rule out any spectral signatures originating from the fluids themselves. The recorded spectra do not contain any prominent Raman bands originating from the intravenous fluids. However, in the case of PAS and plasmalyte-A, a weak Raman band was observed within the 924 cm^{-1} region. All these spectra were acquired using identical parameters for recording Raman spectra of platelets given in the following sections, including an exposure time of 60 seconds, an accumulation number of 2, laser power of 10 mW, and a microscope objective with a magnification of 100x. It has been confirmed that platelets suspended in PAS, Plasmalyte-A, NS 0.9%, NS 3%, and NS 0.45% exclusively exhibit Raman bands originating from the optically trapped platelets.

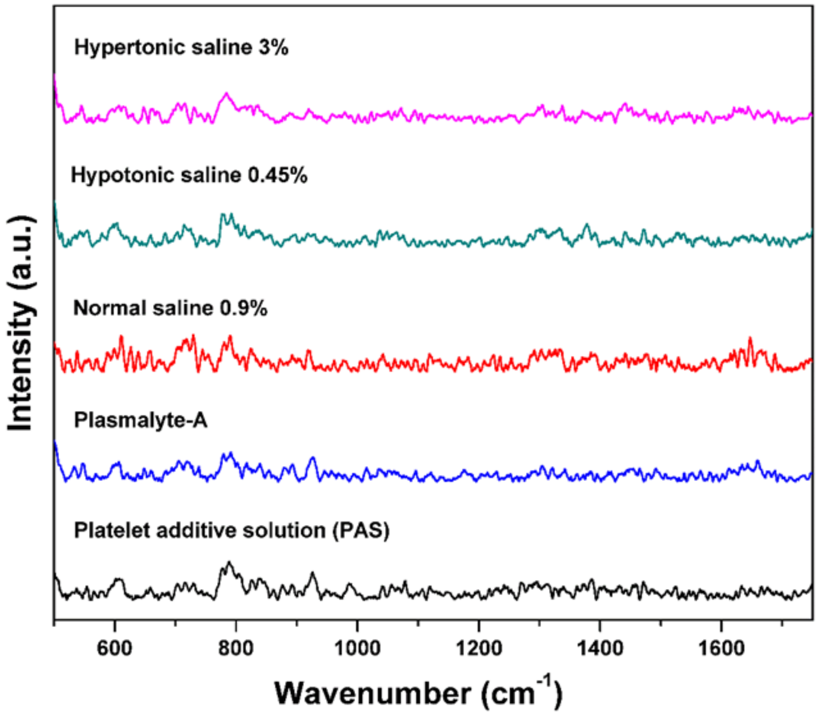

Figure 2S. The Micro-Raman spectra of different intravenous fluids

3. Raman band assignments

Table 1S: The Raman band assignments for the peaks that show intensity variations while comparing platelets in PAS with those treated with intravenous fluids [4-7].

Sl. No.	Wavenumber (cm^{-1})	Vibration	Components	Plasmalyte-A	NS 0.9%	NS 0.45%	NS 3%
				Raman band intensity			
1	618	C-C twist	Phenylalanine	No change	↓	↓	↓
2	653	--	Tyrosine	↓	↓	↓	↓
3	757	Phosphate diester stretch	Phosphatidylethanolamine	No change	↑	↑	↑
4	1042	C-N stretch	Polypeptide	↓	↓	↓	↑
5	1082	C-N stretch	Phospholipid	No change	↑	↑	↑
6	1097	C-C gauche stretch	Phospholipid	↑	↑	↑	↑
7	1121	C-C trans stretch	Phospholipid	↑	↑	↑	↑
8	1152	C-C stretch	β-Carotene	↓	↑	↑	↑
9	1436	CH_2 bend	Phospholipid	No change	↑	↑	↑
10	1445	CH_2 bend	Phospholipid	No change	↑	↑	↑
11	1455	CH_2 bend	Lipids	↓	↑	↑	↑
12	1519	C=C stretch	β-Carotene	↓	↓	↓	↑
13	1653	C=C stretch	Protein, Cholesterol, Amide I	↓	No change	↓	↑

Note: ↑- Increased intensity, ↓- Decreased intensity

4. Platelet separation procedure:

Whole blood units obtained by a smooth venipuncture with continuous blood flow are used to obtain platelet concentrates. The components are separated using the buffy coat method within 6 to 8 hours of collection, and centrifugation is carried out at 22°C. The collected blood was centrifuged at 3300 rpm for 9 minutes at 22°C in order to separate the plasma and red blood cells. Buffy coat method retains the platelets, white blood cells, a small volume of plasma and a little bit of red cells in the buffy coat bag. The buffy coat bag is then let to rest for two to four hours at room temperature after being hanged from a stand at a height. Following this, the buffy coat bags are placed vertically in centrifuge buckets. Diagonally opposite buckets are weighed and adjusted identically to guarantee balance. A second gentle spin at 850 rpm for seven minutes at 22°C is subsequently applied. After centrifugation, the platelet-containing supernatant plasma is moved to the platelet satellite bag, making sure that there is no red cell contamination.

5. Platelet activation mechanism

The structure/morphology of platelets changes significantly upon activation. While inactive platelets have a discoid shape, activation triggers cytoskeletal remodeling, resulting in a spherical form with extended projections like filopodia [8]. Filopodia formation is driven by actin polymerization and regulated by proteins such as Rho GTPases (RhoA, Rac1, and Cdc42) and the Arp2/3 complex. Filopodia have a crucial role in enhancing platelet adhesion and enabling interactions with other platelets, endothelial cells, or components of the subendothelial matrix. An increase in intracellular calcium levels is essential for platelet activation, driving granule secretion, cytoskeletal remodeling, and the externalization of phosphatidylserine (PS). As Ca2+ levels rise, peripheral microtubule coils expand and fold inward toward the cell center, causing activated platelets to undergo transition from a discoid to a spherical shape [9].

Platelet activation at the intracellular and molecular levels encompasses calcium flux, the translocation of negatively charged phospholipids, granule secretion, and undergoes morphological changes [10]. Platelet calcium flux is primarily induced by agonists via “G protein-coupled receptors (GPCRs) or Immunoreceptor Tyrosine-based Activation Motif-linked Receptors” [10]. “Thrombin, ADP, and thromboxane A2 (TxA2) receptors belong to the GPCR family and signal through phospholipase C (PLC) β. ILRs, such as Glycoprotein VI (GPVI) and C-type lectin-like receptor 2 (CLEC-2), modulate Phospholipase C gamma (PLCγ) isoforms” [11, 12]. Both pathways lead to the production of “inositol 1, 4, 5-trisphosphate (IP3)”, which interacts with the “inositol phosphate-sensitive (IPS)” receptor on dense tubules, triggering the release of Ca^{2+} and elevating cytoplasmic Ca^{2+} levels [13]. This rise in Ca^{2+} subsequently activates the cytoskeletal system and governs various cellular processes, including phospholipid translocation, granule secretion, changes in cell shape, and the trafficking, redistribution, and activation of proteins [13].

Negatively charged phospholipids move from the inner leaflet to the outer membrane surface upon platelet activation. By promoting the contact between platelets and the coagulation enzyme complex, which activates serine proteases and triggers thrombin activation, this negatively charged

surface improves coagulation [14]. Activated platelets release lysosomes, dense granules, and α-granules, which help with hemostasis, coagulation, inflammation, vasoconstriction, and angiogenesis [15, 16]. Important substances are released into the extracellular microenvironment, including "growth factors, fibrinogen, and von Willebrand factor (vWF)". In the meanwhile, other components, such as "integrin αIIbβ3, GPVI, GPIb-IX-V complex elements, and P-selectin", are re-distributed or integrated into the cytoplasmic membrane, where they are essential for signal transduction[17].

6. Area under the curve

The area under the curve for the phospholipid band at 1121 cm^{-1} of platelets suspended in various intravenous fluids is shown in Figure 3S, and the corresponding bar plot is given in Figure 4S. The area under the curve of the Raman band was less in platelets suspended in PAS, where the platelets are in their inactive form. In the case of saline-treated samples, the area under the curve of the Raman band at 1121 cm^{-1} was found to be large.

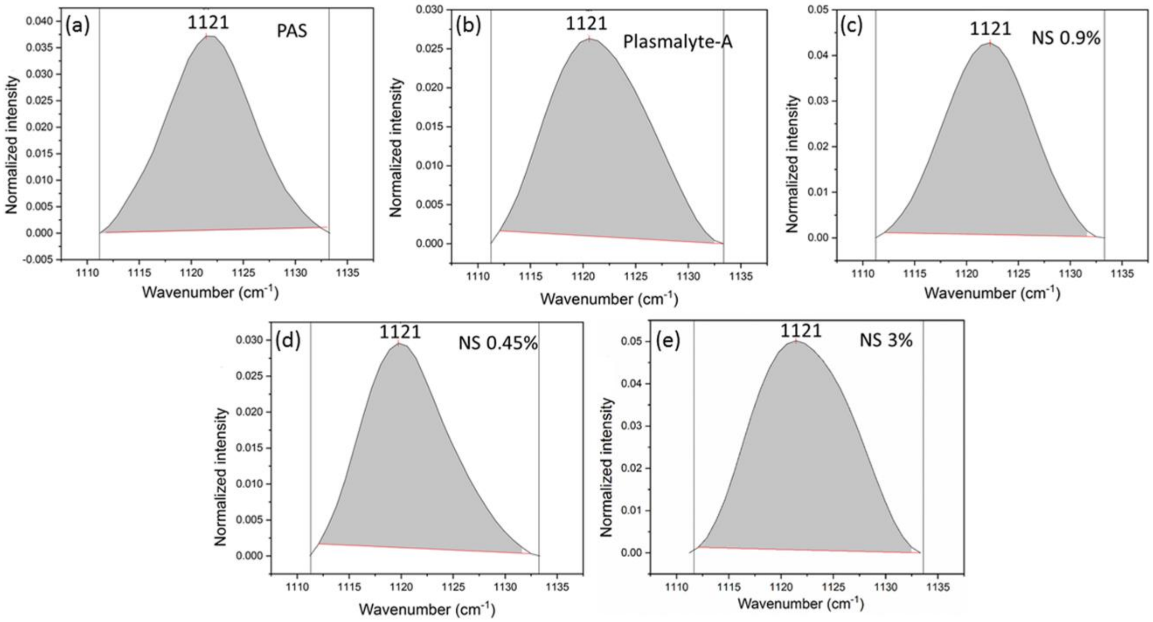

Figure 3S. The area under the curve of Raman peak at 1121 cm^{-1} in different intravenous fluids: (a) PAS (b) Plasmalyte-A (c) 0.9% NS (d) 0.45 % NS and (e) 3% NS

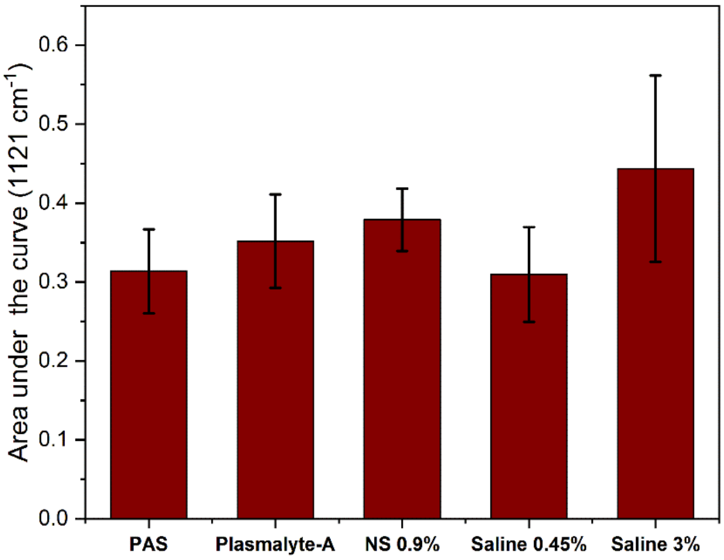

Figure 4S. Bar plot showing area under the curve of Raman peak at 1121 cm^{-1} of platelet in different intravenous fluids

In the case of phospholipids, the bands at 1436 cm^{-1}, 1445 cm^{-1}, and 1455 cm^{-1} have a large area under the curve for the platelets in 3% saline. Supplementary Figure 5S shows the area under the curve plot for Raman bands at 1436 cm^{-1}, 1445 cm^{-1}, and 1455 cm^{-1}. Supplementary Figure 6S shows the bar diagram of these Raman bands. The area under the curve was determined for the Raman bands in the range of 1422 cm^{-1} to 1480 cm^{-1}, which includes all three bands mentioned above.

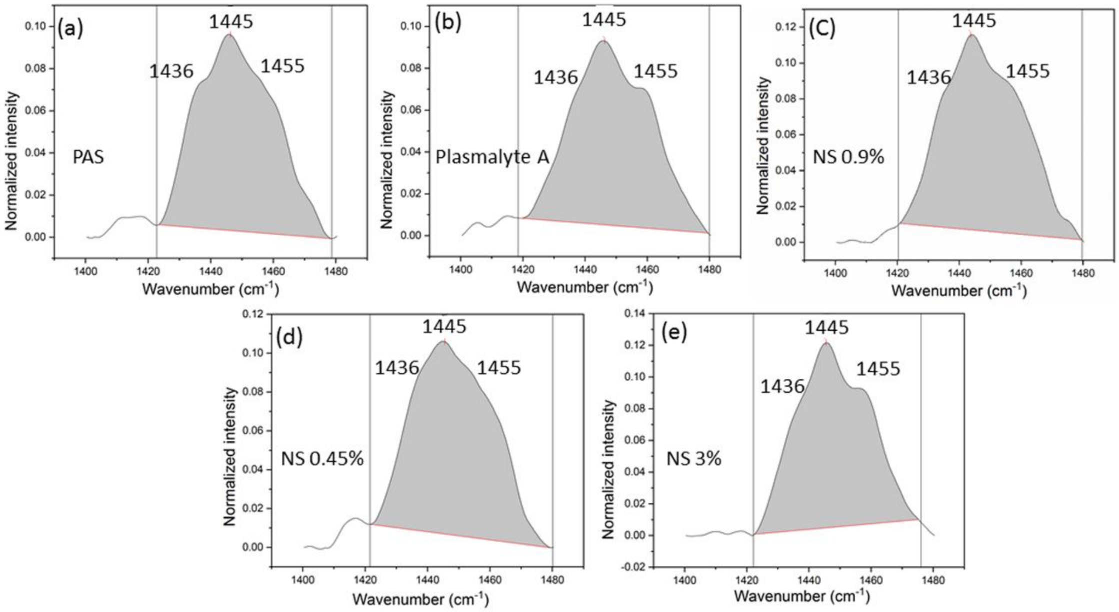

Figure 5S. Area under the curve of Raman bands of platelets at 1436 cm^{-1}, 1445 cm^{-1}, and 1455 cm^{-1} in different intravenous fluids (a) PAS (b) Plasmalyte-A (c) 0.9% NS (d) 0.45 % NS and (e) 3% NS.

Analysis of the bar plot indicates that the area under the curve corresponding to the bands at 1436 cm^{-1}, 1445 cm^{-1}, and 1455 cm^{-1} is significantly larger in platelets treated with 3% NS. This observation suggests a clear increase in phospholipid content on the surface of the activated platelets. Similarly, platelets treated with 0.45% NS and 0.9% NS also demonstrate a substantial area under the curve in comparison to those suspended in PAS. Conversely, platelets in plasmalyte A exhibit comparable areas under the curve in this range.

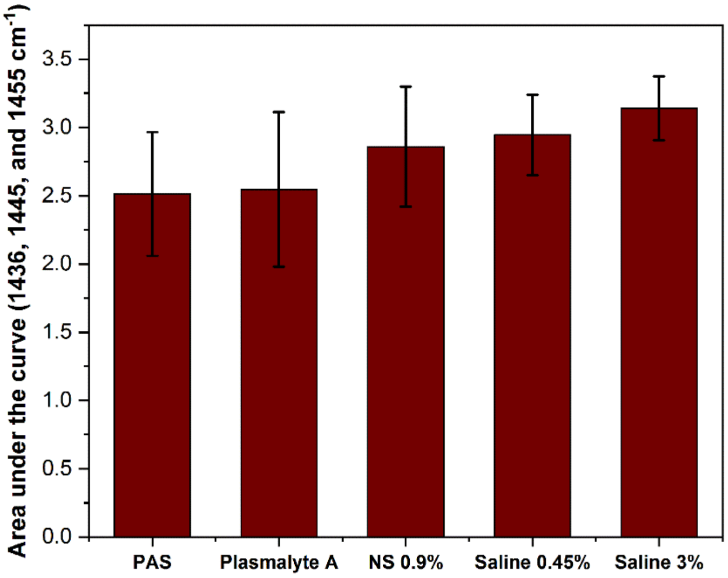

Figure 6S. Bar plot showing area under the curve of Raman bands at 1436 cm^{-1}, 1445 cm^{-1}, and 1455 cm^{-1} of platelet in different intravenous fluids.

7. The deconvolution of Amide I band (1600-1700 cm^{-1})

The unfolding or structural rearrangement of the polypeptide chain occurs during protein denaturation and aggregation, resulting in modifications to the content of α-helices and β-sheets. [18, 19]. The vibrational modes of peptide bonds are altered by these structural alterations, especially in amide I (about 1600–1700 cm^{-1}). Intramolecular hydrogen bonding (C=O ··· H-N) along the polypeptide backbone stabilizes the α-helix structure. Mechanical, chemical, or thermal stress can disturb the hydrogen bond. Therefore, the α-helix intramolecular hydrogen bonds may be weakened or broken under these circumstances. The molecular disarray and subsequent transformation of the structure into a random coil or β-sheet may also occur during this process.

Multiple peak fitting of the amide I band was performed using Origin software with the Gaussian model. The ratio of Raman band areas for α-helix and β-sheet exhibits differences in the spectra of the platelets suspended in PAS compared to those in other intravenous fluids. As key components of a protein's secondary structure, changes in α-helix and β-sheet proportions are indicative of structural modifications. An increase in β-sheet band area alongside a reduction in α-helix intensity suggests protein denaturation or aggregation conformational changes [19, 20].

After curve deconvolution, some additional Raman bands are observed in the 1600 – 1700 cm^{-1} region [21] (Figure 7S a, b, c, d, and e). In PAS, "1622 cm^{-1} (β-sheet), 1633 cm^{-1} (β-sheet), 1641 cm^{-1} (random coil), 1654 cm^{-1} (α-helix), 1669 cm^{-1} (β-sheet), and 1678 cm^{-1} (β-sheet)" are the additional bands obtained after curve resolution [22-26]. In the case of platelets suspended in plasmalyte-A, the curve resolution shows seven closely resolved bands, which include "1617 cm^{-1} (Tyrosine, β-turn), 1644 cm^{-1} (random coil), 1649 cm^{-1} (random coils), 1671 cm^{-1} (β-sheet), and 1686 cm^{-1} (β- turn)" [22, 24, 25]. In the 1655 to 1660 cm^{-1} region, there are two closely overlapped bands observed at 1655 cm^{-1}, and 1658 cm^{-1}, according to the literature, these bands are assigned to "α-helix" [24]. The platelets suspended in 0.9% NS have Raman bands at "1624 cm^{-1} (β-sheet), 1647 cm^{-1} (random coil), 1649 cm^{-1} (random coils), 1655 cm^{-1} (α-helix), 1664 cm^{-1} (β- sheet), 1675 cm^{-1} (β-sheet) and 1686 cm^{-1} (β- turn)". The platelets in 0.45% NS have Raman bands at "1622 cm^{-1} (β-sheet), 1642 cm^{-1} (random coil), 1653 cm^{-1} (α-helix), 1662 cm^{-1} (β-sheet), 1675 cm^{-1} (β-sheet), and 1681 cm^{-1} (β- turn)". In 3% NS, the amide I region of platelets has curve-resolved bands at "1618 cm^{-1} (Tyrosine, β-turn), 1630 cm^{-1} (β-sheet), 1641 cm^{-1} (random coil), 1655 cm^{-1} (α-helix), 1669 cm^{-1} (β- sheet), and 1678 cm^{-1} (β-sheet)".

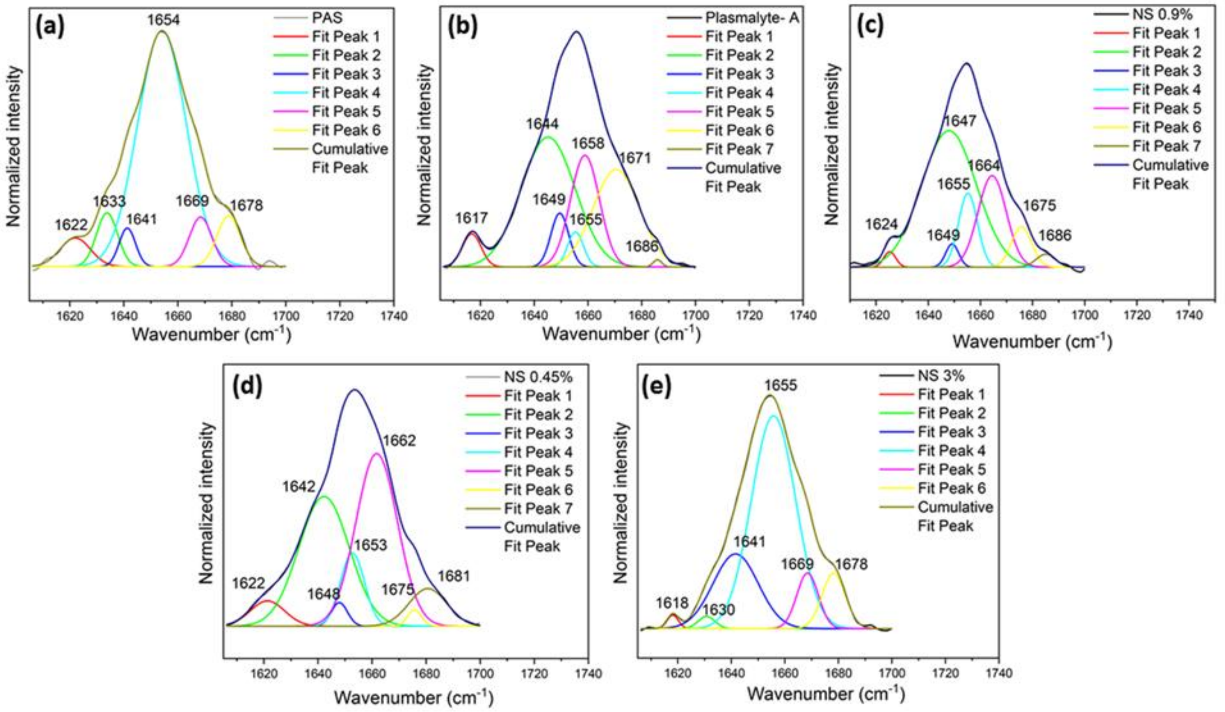

Figure 7S. Curve resolved amide I (1600 – 1700 cm^{-1}) Raman bands of platelet in different intravenous fluids: (a) PAS (b) Plasmalyte A, (c) 0.9% NS (d) 0.45% NS, and (e) 3% NS.

The ratio of the area under the curve for α-helix and β-sheet, as presented in Table 2S, was large in platelets suspended in PAS, suggesting a low probability of protein denaturation, aggregation or structural alterations. Conversely, in other IV fluids, the ratio was lower, indicating an increased probability of protein denaturation or structural changes. The reason for these changes might be due to the change in salinity and pH variations.

Table 2S. The ratio of the area under the curve for α-helix, β-sheet and random coil

Intravenous fluids	Ratio of the areas (~1655 /~1669 cm^{-1})	Ratio of the areas (~1655 /~1678 cm^{-1})	Ratio of the areas (~1655 /~1641 cm^{-1})
PAS	10.3365	9.6512	18.2665
Plasmalyte - A	0.8387	-	0.5401
NS (0.9%)	0.5408	1.7163	0.1869
NS (0.45%)	0.2447	7.775	0.2672
NS (3%)	7.8974	7.2891	2.8059

8. References

(1) van der Meer, P. F.; de Korte, D. Platelet additive solutions: a review of the latest developments and their clinical implications. *Transfus. Med. Hemother.* **2018**, *45* (2), 98-102.
(2) Van der Meer, P. PAS or plasma for storage of platelets? A concise review. *Transfus. Med.* **2016**, *26* (5), 339-342.
(3) de Wit, Y. E.; Vlaar, R.; Gouwerok, E.; Hamzeh-Cognasse, H.; van Mierlo, G.; Bulder, I.; Lagerberg, J. W.; de Korte, D.; Cognasse, F.; Ten Brinke, A. Platelet concentrates in platelet additive solutions generate less complement activation products during storage than platelets stored in plasma. *Blood transfusion* **2023**, *21* (2), 157.
(4) Barkur, S.; Bankapur, A.; Chidangil, S.; Mathur, D. Effect of infrared light on live blood cells: Role of β-carotene. *J. Photochem. Photobiol., B* **2017**, *171*, 104-116.
(5) Czamara, K.; Majzner, K.; Pacia, M. Z.; Kochan, K.; Kaczor, A.; Baranska, M. Raman spectroscopy of lipids: a review. *J. Raman Spectrosc.* **2015**, *46* (1), 4-20.
(6) Giancaspro, J.; Scollan, P.; Rosario, J.; Miller, E.; Braziel, S.; Lee, S. Structural determination of model phospholipid membranes by Raman spectroscopy: Laboratory experiment. *Biochem. Mol. Biol. Educ.* **2022**, *50* (2), 181-192.
(7) Zhu, G.; Zhu, X.; Fan, Q.; Wan, X. Raman spectra of amino acids and their aqueous solutions. *Spectrochim. Acta, Part A* **2011**, *78* (3), 1187-1195.
(8) Thomas, S. G. The structure of resting and activated platelets. *Platelets* **2019**, 47-77.
(9) Patel-Hett, S.; Richardson, J. L.; Schulze, H.; Drabek, K.; Isaac, N. A.; Hoffmeister, K.; Shivdasani, R. A.; Bulinski, J. C.; Galjart, N.; Hartwig, J. H. Visualization of microtubule growth in living platelets reveals a dynamic marginal band with multiple microtubules. *Blood,* **2008**, *111* (9), 4605-4616.
(10) Tang, L.; Liu, C.; Rosenberger, P. Platelet formation and activation are influenced by neuronal guidance proteins. *Front. Immunol.* **2023**, *14*, 1206906.
(11) Rayes, J.; Watson, S. P.; Nieswandt, B. Functional significance of the platelet immune receptors GPVI and CLEC-2. *J. clin. invest.* **2019**, *129* (1), 12-23.
(12) Offermanns, S. Activation of platelet function through G protein–coupled receptors. *Circ. Res.* **2006**, *99* (12), 1293-1304.
(13) Rubenstein, D. A.; Yin, W. Platelet-activation mechanisms and vascular remodeling. *Compr. Physiol.* **2011**, *8* (3), 1117-1156.
(14) Lhermusier, T.; Chap, H.; Payrastre, B. Platelet membrane phospholipid asymmetry: from the characterization of a scramblase activity to the identification of an essential protein mutated in Scott syndrome. *J. Thromb. Haemostasis* **2011**, *9* (10), 1883-1891.

(15) Abou-Saleh, H.; Théorêt, J.-F.; Yacoub, D.; Merhi, Y. Neutrophil P-selectin-glycoprotein-ligand-1 binding to platelet P-selectin enhances metalloproteinase 2 secretion and platelet-neutrophil aggregation. *Thromb. Haemostasis* **2005**, *94* (12), 1230-1235.
(16) Lam, F. W.; Da, Q.; Guillory, B.; Cruz, M. A. Recombinant human vimentin binds to P-selectin and blocks neutrophil capture and rolling on platelets and endothelium. *J. Immunol.* **2018**, *200* (5), 1718-1726.
(17) Nieswandt, B.; Watson, S. P. Platelet-collagen interaction: is GPVI the central receptor? *Blood* **2003**, *102* (2), 449-461.
(18) Di Gregorio, E.; Staelens, M.; Hosseinkhah, N.; Karimpoor, M.; Liburd, J.; Lim, L.; Shankar, K.; Tuszyński, J. A. Raman Spectroscopy Reveals Photobiomodulation-Induced α-Helix to β-Sheet Transition in Tubulins: Potential Implications for Alzheimer's and Other Neurodegenerative Diseases. *Nanomaterials* **2024**, *14* (13), 1093.
(19) Litvinov, R. I.; Faizullin, D. A.; Zuev, Y. F.; Weisel, J. W. The α-helix to β-sheet transition in stretched and compressed hydrated fibrin clots. *Biophys. J.* **2012**, *103* (5), 1020-1027.
(20) Kuhar, N.; Sil, S.; Verma, T.; Umapathy, S. Challenges in application of Raman spectroscopy to biology and materials. *RSC Adv.* **2018**, *8* (46), 25888-25908.
(21) Pezzotti, G. Raman spectroscopy in cell biology and microbiology. *J. Raman Spectrosc.* **2021**, *52* (12), 2348-2443.
(22) Kuhar, N.; Sil, S.; Umapathy, S. Potential of Raman spectroscopic techniques to study proteins. *Spectrochim. Acta, Part A* **2021**, *258*, 119712.
(23) Carmona, P.; Molina, M.; López-Tobar, E.; Toledano, A. Vibrational spectroscopic analysis of peripheral blood plasma of patients with Alzheimer's disease. *Anal. Bioanal. Chem.* **2015**, *407*, 7747-7756.
(24) García-Rubio, D. L.; de la Mora, M.; Badillo-Ramírez, I.; Cerecedo, D.; Saniger, J. M.; Benítez-Benítez, J. L.; Villagrán-Muniz, M. Analysis of platelets in hypertensive and normotensive individuals using Raman and Fourier transform infrared-attenuated total reflectance spectroscopies. *J. Raman Spectrosc.* **2019**, *50* (4), 509-521.
(25) Wu, K.; Li, Y.; Chen, J. Effect of pH on the Structure, Functional Properties and Rheological Properties of Collagen from Greenfin Horse-Faced Filefish (Thamnaconus septentrionalis) Skin. *Mar. Drugs* **2024**, *22* (1), 45.
(26) Li, Y.; Yang, L.; Wu, S.; Chen, J.; Lin, H. Structural, functional, rheological, and biological properties of the swim bladder collagen extracted from grass carp (Ctenopharyngodon idella). *Lwt* **2022**, *153*, 112518.
